# Antidepressant Effect of Dimeric Dipeptide GSB-106, an Original Low-Molecular-Weight Mimetic of BDNF

**Published:** 2013

**Authors:** S.B. Seredenin, T.A. Voronina, T.A. Gudasheva, T.L. Garibova, G.M. Molodavkin, S.A. Litvinova, E.A. Elizarova, V.I. Poseva

**Affiliations:** V.V.Zakusov Institute of Pharmacology, Russian Academy of Medical Sciences, Baltiyskaya Str., 8, Moscow, Russia, 125315

**Keywords:** BDNF, mimetic, GSB-106, antidepressant activity, forced swimming test, tail suspension test

## Abstract

A large amount of clinical and experimental data suggest the involvement of neurotrophins, in particular the brain-derived neurotrophic factor (BDNF), in depression pathogenesis. However, the therapeutic use of BDNF is limited because of its instability in biological fluids, poor blood-brain barrier (BBB) permeability, and the presence of side effects. A low-molecular-weight mimetic GSB-106, which is a substituted dimeric dipeptide *bis*(N-monosuccinyl-L-seryl-L-lysine)hexamethylenediamide, was designed and synthesized based on the BDNF fourth loop structure at the V.V. Zakusov Institute of Pharmacology (RAMS). GSB-106 was found to exhibit an antidepressant activity in various models of depressive-like state when administered intraperitoneally to outbred mice and rats. An effect for the substance, when administered daily for 4–5 days, was detected in the Porsolt forced swimming test (0.1 and 1.0 mg/kg) and in the tail suspension test in mice (1.0 and 1.5 mg/ kg). An effect for GSB-106 at doses of 0.1 and 0.5 mg/kg was observed after a single application in experiments on rats in the Nomura water wheel test. The obtained evidence supports the hypothesis on the involvement of BDNF in the pathogenesis of various depression conditions, thus opening prospects for searching for new original antidepressants.

## INTRODUCTION

According to the WHO, 4–5% of the world population suffers from depression and depressions could become the most prevalent disease by 2030 [1, 2]. Even now about 20% of mental patients in economically developed countries suffer from endogenous and psychogenic depressive disorders [3].

Disregulation of the major monoaminergic systems of the brain, including the serotonergic, noradrenergic, and dopaminergic ones, has for a long time been regarded as the primary pathophysiological mechanism for the development of depressive disorders. The application of virtually all antidepressants that are being currently used, which are either monoamine oxidase (MAO) or monoamine reuptake inhibitors, does not always yield the desired clinical results.

A large body of evidence for the important role of the changes in the neurotrophin level, BDNF especially, in depression pathogenesis has been accumulated over the past decades [4-6]. Clinical studies have shown that the BDNF blood content in patients with severe depression is significantly reduced and recovers after the administration of antidepressants [7, 8].

Based on depression models, BDNF has been shown to exhibit a pronounced antidepressant effect upon central administration [9, 10]. The high resistance of transgenic mice with elevated levels of this neurotrophin to depression also provides evidence of the antidepressant properties of BDNF [11]. In addition, positive feedback between BDNF and serotonin was found in [12].

The therapeutic use of BDNF is limited by its instability in biological fluids, poor blood-brain barrier permeability, the risk of a reaction, and side effects due to its pleiotropy.

In connection with this, the strategy to develop new compounds on the basis of low-molecular-weight mimetics of BDNF, which would possess an antidepressant activity when administered systemically and would have none of the side effects typical of the original neurotrophin, seems rather promising. A series of low-molecular-weight mimetics of BDNF has been described. Thus, a group of Australian researchers have designed bicyclic and tricyclic dimeric peptides with agonistic activity on the basis of the second loop [13]. A group of American scientists [14] have obtained seven non-peptide compounds on the basis of the second loop, as well. However, no data have been reported regarding an antidepressant activity for the described mimetics of BDNF.

A low-molecular-weight mimetic GSB-106 [15, 16], which is a substituted dimeric dipeptide bis(N-monosuccinyl- L-seryl-L-lysine)hexamethylenediamide, was designed and synthesized based on the BDNF fourth loop structure at the V.V. Zakusov Institute of Pharmacology (RAMS). GSB-106 was selected in the course of pharmacological screening of four compounds, mimetics of the first and fourth loops of BDNF, as a dimeric dipeptide exhibiting antidepressant activity in the Balb/c mouse line upon single administration in the Porsolt forced swimming test [16].

*In vitro *studies of GSB-106 on a culture of immortalized NT 22 mouse hippocampal cells demonstrated that this compound at concentrations ranging from 10^-5^ to 10^-8^ M exhibits a neuroprotective activity in models of oxidative stress and glutamate toxicity. The neuroprotective activity of GSB-106 was also detected in cultured SH-SY5Y human neuroblastoma cells when treated with neurotoxin 6-hydroxidopamine [17].

The aim of the present work was to study GSB- 106 antidepressant properties on various depressive state models in outbred mice and rats upon single and subchronic administration.

## EXPERIMENTAL

GSB-106 was studied on white outbred male rats (2–2.5 months old, weighing 270–290 g) and male mice weighing 22–25 g received from the “Stolbovaya” Central Laboratory for Animal Breeding (Moscow Region, Russia). Animal husbandry activities were performed in compliance with good laboratory practices regulations and sanitary rules for the maintenance of experimental biological clinics (vivarium). The study was conducted in accordance with Order of the Ministry of Health Care and Social Development of the Russian Federation № 708n of 23.08.2010 “Approval of the Rules of Good Laboratory Practice.” GSB-106 synthesized at the V.V. Zakusov Institute of Pharmacology of RAMS was used in the study.

The antidepressant activity of the compounds was evaluated in the Porsolt forced swimming test [18], the Nomura water wheel test [19], and the tail suspension test in mice [20].

The experimental setup for creating the Porsolt depression-like state (behavioral despair) in mice consisted of a cylindrical vessel (10 cm in diameter and 30 cm high). The vessel was filled with water to a height of 18 cm, and its temperature was maintained at 27oC. Preliminarily, one day prior to testing, each animal was immersed in a container with water for 5–6 min for adaptation. On the day of the experiment, the animal was placed in the vessel with water so that it neither could escape from the vessel nor could find a support within. Once in water, the animals began to show violent motor activity aimed at finding a way out of the aversive stress situation, but then they gave up and hung in the water in a characteristic pose, remaining completely motionless or making the small movements necessary to keep their head above water. This behavior is considered as a sign of desperation, despondency, and a depressive-like state [18]. A measure of the severity of the depressive-like state in this test is immobility duration, i.e. the sum of immobility episodes over 6 min of observation for each animal. A statistically significant reduction in the immobilization duration is considered to be the antidepressant activity criterion.

A four-channel setup designed at the V.V. Zakusov Institute of Pharmacology (RAMS) [21] was used to model a depressive-like state in rats by the method of Nomura [19] in a vessel with water and freely rotating wheels. The setup consisted of a 64 x 30 x 42 cm vessel divided into four equal compartments. Each compartment comprised a 11 cm wide wheel with 12 blades (2 cm wide each); the outer diameter of the wheel was 10 cm. Magnets were anchored on the edges of each wheel, and reed switches were located over the wheels and responded each time when a magnet passed under them. The automatic detection of wheel rotation was carried out in this manner and served as objective measure of animal activity. The vessel was filled with water at 25°C until it reached the midline of the wheel. Rats were placed in each compartment, with their nebs oriented away from the wheel, and the wheel rotation speed was recorded for 10 min with electromechanical counters.

The animals’ tails were tied to a horizontal crossbar in the tail suspension test [20]. First, the animals placed into stressful situations began to show motor activity aimed at finding a way out of the aversive conditions, but then they stopped this activity and hung on the crossbar remaining almost completely immobile.

Dipeptide GSB-106 was dissolved in distilled water and administered to the animals intraperitoneally at doses of 0.01, 0.1, 0.5, 1.0, and 1.5 mg/kg 30 min prior to testing once or repeatedly once a day for 4–5 days. The control animals received normal saline in the same regimen.

Statistical processing of the results was carried out with the Biostatistics III program using the Student’s and Mann–Whitney tests.

## RESULTS AND DISCUSSION

**Antidepressant activity of GSB-106 in the Porsolt forced swimming test in mice**

It was found that immobilization for 238–278 s in different groups of control mice was observed after a period of activity (Table 1). GSB-106, when administered once at doses of 0.1 and 1.0 mg/kg, showed a tendency to reduce the immobilization time (Table 1).

**Table 1:** Antidepressant effect of GSB-106 in mice (by Porslot)

Dose of GSB-106 administered intraperitoneally, mg/kg, once a day	Administration frequency	Immobilization time, s (M ± SEM)
Control (saline)	1	255.61 ± 25.07
0.1	1	206.29 ± 33.35
1.0	1	204.83 ± 26.67
Control (saline)	5	278.38 ± 12.02
0.1	5	231.41 ± 11.22*
Control (saline)	4	271.73 ± 13.37
1.2	4	205.76 ± 11.02*
Control (saline)	1	238.50 ± 15.37
Amitriptyline, 10.0 mg/kg	1	134.62 ± 23.42*

*p < 0.05 – statistical significance of the differences with the Mann-Whitney U test compared to the control group.

GSB-106, when administrated subchronically at a dose of 0.1 mg/kg for five days or at a dose of 1.0 mg/kg for four days, corrected the animal’s behavior in the forced swimming test, significantly reducing the duration of immobilization episodes compared to the control group: by 1.2 times using GSB-106 at a dose of 0.1 mg/kg and by 1.3 times when GSB-106 was administered at a dose of 1.0 mg/kg (Table 1).

Therefore, GSB-106 at doses of 0.1 and 1.0 mg/kg upon repeated administration for 4–5 days exhibited an antidepressant effect in the Porsolt behavioral despair test, which manifested itself in a statistically significant decrease in the immobilization time of the animals. An increase in the antidepressant effect of BDNF upon repeated administration was also described. Thus, BDNF (0.25–1.0 μg) when bilaterally injected once into the hippocampus reduced the immobilization duration twofold [10], and when infused in the midbrain of rats for 4–5 days at a dose of 12–24 μg/day it reduced the immobilization duration threefold in the Porsolt forced swimming test [9].

**Antidepressant effect of GSB-106 in the Nomura depressive-like state test in rats**

The rats in the control group were found to make on average 87 wheel turns during 5 min of registration (Figure). GSB-106 at a dose of 0.01 mg/kg did not cause any increase in the number of wheel turns, but when administered at a higher dose (0.1 mg/kg), the compound showed a distinct antidepressant activity as evidenced by a statistically significant increase (1.8 times) in the number of wheel turns made by the rats compared to the parameters of the control animals (Figure). The antidepressant effect of GSB-106 at a dose of 0.5 mg/kg was stronger, and the number of wheel turns made by the rats increased twofold. However, with a further increase in the dose of GSB-106 to 1.0 mg/kg its antidepressant effect decreased and the number of wheel turns remained the same as that in the control animals (Figure).

**Figure:**
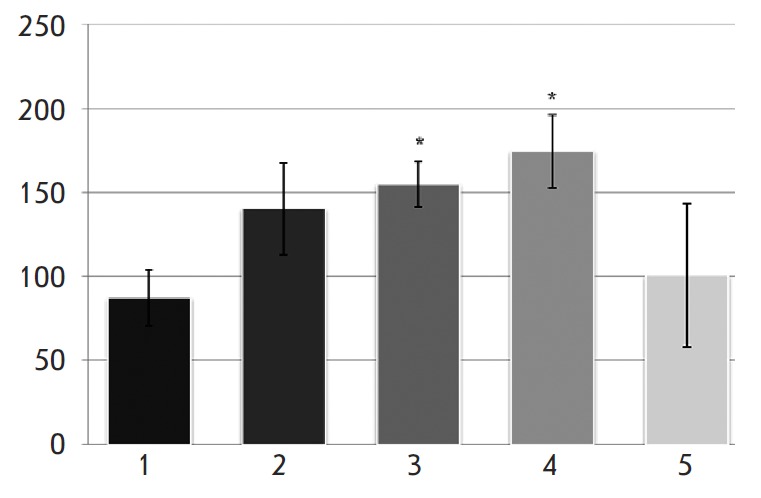
Antidepressant effect of GSB-106 in the Nomura depressive- like state test. Dose of intraperitoneal administration of GSB-106, mg/kg: 1 – control; 2 – 0.01; 3 – 0.1; 4 – 0.5; 5 – 1.0. Y-direction: the number of wheel turns *p < 0.05 statistical significance of the deviation from the control group with the Mann-Whitney U-test

Hence, GSB-106 at doses of 0.1 and 0.5 mg/kg produced a distinct antidepressant effect in the Nomura forced swimming test. The plot of the effect *vs *dose of GSB-106 is bell-shaped.

**Antidepressant activity of GSB-106 in the depressivelike state test caused by suspending mice by the tail**

It was found that the average immobilization time upon suspension by the tail in the control group of animals was 174 and 148 s for different groups. GSB-106, when administered subchronically (4 days) and intraperitoneally at doses of 0.1 and 0.5 mg/kg, did not alter the animal’s immobilization duration in this test compared to the control. However, GSB-106 had a clear antidepressant effect at higher doses. GSB-106 at doses of 1.0 and 1.5 mg/kg (4 days, intraperitoneally) significantly (p=0.04) decreased (1.3 times) the immobilization time in mice in the tail suspension test (Table 2).

**Table 2:** Antidepressant effect of GSB-106 upon subchronic (4 days) administration in the depressive-like state test in mice caused by tail suspension

Dose of GSB-106 administered intraperitoneally, mg/kg	Immobilization time, s (M ± SEM)
Control (saline)	174.00 ± 10.4
0.1	145.20 ± 15.81
1.0	135.50 ± 12.85*
Control (saline)	148.25 ± 6.38
0.5	126.22 ± 9.89
1.5	120.13 ± 10.53*

*Statistical significance of the deviation from the control, p ≤ 0.05 (Student’s t-test).

Thus, the antidepressant effect of the GSB-106 dipeptide was clearly revealed under conditions of three validated methods for modeling the depressivelike state: in the Porsolt behavioral despair test (0.1 and 1.0 mg/kg, 4–5 days), in the Nomura water wheel test (0.1 and 0.5 mg/kg, single dose), and in the Steru tail suspension test in mice (1.0 and 1.5 mg/kg, 4 days).

It is important that the antidepressant effect of GSB-106 was observed upon systemic intraperitoneal administration to outbred mice and rats both as a single dose and as repeated daily doses in the range of 0.1–1.5 mg/kg. It appears that the stronger pronounced effect of GSB-106 in rats is related to species differences and to the methodological features of the evaluation.

As mentioned above, according to the neutrophin theory of depression development, low BDNF levels in the central nervous system damage brain structures and cause the development of depressive states; however, the use of antidepressants or administration of BDNF to animals corrects these disorders. The antidepressant effect of GSB-106 attained in the present work is similar to that of BDNF upon intraventricular infusion or upon administration of the latter to the brain regions of an animal responsible for depression [8-10]. In the study by Schmidt and Duman [22], systemic (subcutaneous) administration of recombinant BDNF to mice caused an antidepressant effect characterized by a 1.5-fold decrease in the immobilization duration in the forced swimming test. However, this effect of BDNF was only observed when used at doses 6–7 times higher than those of GSB-106, and only after long-term administration (for 7–14 days). Induction of neurogenesis in the hippocampus and midbrain was a functional consequence of the antidepressant action of recombinant BDNF; the authors attributed its mechanism to the increased BDNF level and to the increased level of activation/phosphorylation of ER K and CRE B in the downstream targets of the BDNF-TrkB signaling pathways [22]. Previously, we found that GSB-106, the mimetic of BDNF, activates TrkB and its ER K and the AKT signaling pathways [23] involved in neuronal survival and this fact could presumably underly its antidepressant effect. Moreover, the ability of the GSB-106 dipeptide dimer to phosphorylate TrkB was selective, since no neuroprotective activity of GSB-106 was found in aPC12 cell line not expressing the full-length TrkB but expressing other neurotrophin receptors [23].

On one hand, the resulting data on the antidepressant activity of GSB-106, the low molecular weight mimetic of BDNF, support the hypothesis regarding the involvement of BDNF in the pathogenesis of various depressive states, while on the other hand opening prospects for designing a novel antidepressant (original in its structure and mechanism of action) based on the newly synthesized compound.

## Abbreviations

MAO: monoamine oxidase
BDNF: brain-derived neurotrophic factor
GSB: 106 – bis(N-monosuccinyl-L-seryl-L-lysine)hexamethylenediamide
TrkB: tropomyosin-related receptor kinase B
AKT: serine/threonine protein kinase
CREB: cAMP response element binding protein
ERK: extracellular signalregulated kinase
